# Nine new species of the spider genus *Pireneitega* Kishida, 1955 (Agelenidae, Coelotinae) from Xinjiang, China

**DOI:** 10.3897/zookeys.601.7893

**Published:** 2016-06-29

**Authors:** Xiaoqing Zhang, Zhe Zhao, Guo Zheng, Shuqiang Li

**Affiliations:** 1 College of Life Sciences, Shenyang Normal University, Shenyang, Liaoning 110034, China; 2 Institute of Zoology, Chinese Academy of Sciences, Beijing 100101, China

**Keywords:** Taxonomy, description, diagnosis, Central Asia, Paracoelotes

## Abstract

Nine new *Pireneitega* species collected from Xinjiang, China are described as new to science: *Pireneitega burqinensis* **sp. n.** (♂♀), *Pireneitega fuyunensis* **sp. n.** (♂♀), *Pireneitega gongliuensis* **sp. n.** (♂♀), *Pireneitega huochengensis* **sp. n.** (♂♀), *Pireneitega lini* **sp. n.** (♀), *Pireneitega liui* **sp. n.** (♂♀), *Pireneitega wensuensis* **sp. n.** (♂), *Pireneitega wui* **sp. n.** (♂) and *Pireneitega yaoi* **sp. n.** (♀). DNA barcodes were obtained for all these species for future use.

## Introduction

The spider genus *Pireneitega* was established by Kishida (1955). Its type species is *Amaurobius roscidus* C.L. Koch, 1843 from Germany, considered to be a junior synonym of *Pireneitega segestriformis* (Dufour, 1820). *Pireneitega* was for a long time regarded as a *nomen nudum* until Wang and Jäger (2007) found reasons to revalidate this name and to make *Paracoelotes* Brignoli, 1982 its junior synonym. Currently, there are twenty-one valid *Pireneitega* species, distributed widely from the Iberian Peninsula to Japan and Sakhalin; eleven of them are known from East Asia, six are known from Central Asia, and other four from Europe (Li and Lin 2015, World Spider Catalog 2016). This paper provides descriptions of nine new *Pireneitega* species collected from Xinjiang in northwestern China.

## Material and methods

Specimens were examined with a Leica M205C stereomicroscope. Images were captured with an Olympus C7070 wide zoom digital camera (7.1 megapixels) mounted on an Olympus SZX12 dissecting microscope. Epigynes and male palps were examined after dissection from the spiders’ bodies. The epigyne was cleared by boiling it in a 10% KOH solution before taking photos of the vulva.

All measurements were obtained using a Leica M205C stereomicroscope and are given in millimeters. Leg measurements are given as: Total length (femur, patella + tibia, metatarsus, tarsus). Only structures (palp and legs) of the left side of the body are described and measured. The terminology used in the text and the figure legends follows Wang (2002). Abbreviations used in this paper and in the figure legends are: A = epigynal atrium; ALE = anterior lateral eye; AME = anterior median eye; AME-ALE = distance between AME and ALE; AME-AME = distance between AME and AME; ALE-PLE = distance between ALE and PLE; CD = copulatory duct; CF = cymbial furrow; CO = conductor; E = embolus; EB = embolic base; ET = epigynal tooth; FD = fertilization duct; H = epigynal hood; MA = median apophysis; PA = patellar apophysis; PLE = posterior lateral eye; PME = posterior median eye; PME-PLE = distance between PME and PLE; PME-PME = distance between PME and PME; R = receptacle; RTA = retroventral tibial apophysis; ST = subtegulum; T = tegulum; TC = tip of conductor.

DNA barcodes were obtained for future use. A partial fragment of the mitochondrial gene cytochrome oxidase subunit I (COI) was amplified and sequenced for nine new species and one old species using Primers LCO1490-oono (5’-CWACAAAYCATARRGATATTGG-3’) (Folmer et al. 1994; Miller et al. 2010) and HCO2198-zz (5’-TAAACTTCCAGGTGACCAAAAAATCA-3’) (Folmer et al. 1994; Chen et al. 2015). For additional information on extraction, amplification, and sequencing procedures, see Zhao et al. 2013. All sequences were deposited in GenBank and the accession numbers are provided in Table 1.

**Table 1.** Voucher specimen information.

Species	GenBank accession number	Sequence length	Collection localities
*Pireneitega burqinensis* sp. n.	KX011867	630bp	China: Xinjiang: Burqin
*Pireneitega fuyunensis* sp. n.	KX011859	630bp	China: Xinjiang: Fuyun
*Pireneitega gongliuensis* sp. n.	KX011862	630bp	China: Xinjiang: Gongliu
*Pireneitega huochengensis* sp. n.	KX011861	630bp	China: Xinjiang: Huocheng
*Pireneitega lini* sp. n.	KX011865	630bp	China: Xinjiang: Akto
*Pireneitega liui* sp. n.	KX011860	630bp	China: Xinjiang: Xinyuan
*Pireneitega tianchiensis* (Wang, Yin, Peng & Xie, 1990)	KX011858	630bp	China: Xinjiang: Changji
*Pireneitega wensuensis* sp. n.	KX011864	630bp	China: Xinjiang: Wensu
*Pireneitega wui* sp. n.	KX011866	630bp	China: Xinjiang: Kizilsu
*Pireneitega yaoi* sp. n.	KX011863	630bp	China: Xinjiang: Hoboksar

All of the specimens (including molecular vouchers) are deposited in the Institute of Zoology, Chinese Academy of Sciences
(IZCAS) in Beijing, China.

## Taxonomy

### Family Agelenidae C.L. Koch, 1837 Subfamily Coelotinae F.O. P.-Cambridge, 1893

#### Genus Pireneitega

Taxon classificationAnimaliaAraneaeAgelenidae

Kishida, 1955

Pireneitega Kishida, 1955: 21. Type species Amaurobius roscidus C.L. Koch, 1843 (=Pireneitega segestriformis Dufour, 1820) from Germany; Wang and Jäger 2007: 46 (synonymized 2 genera).
Paracoelotes Brignoli, 1982: 348. Type species Coelotes armeniacus Brignoli, 1978 from Turkey; Wang 2002: 112.

##### Diagnosis.

**Figure 1.**
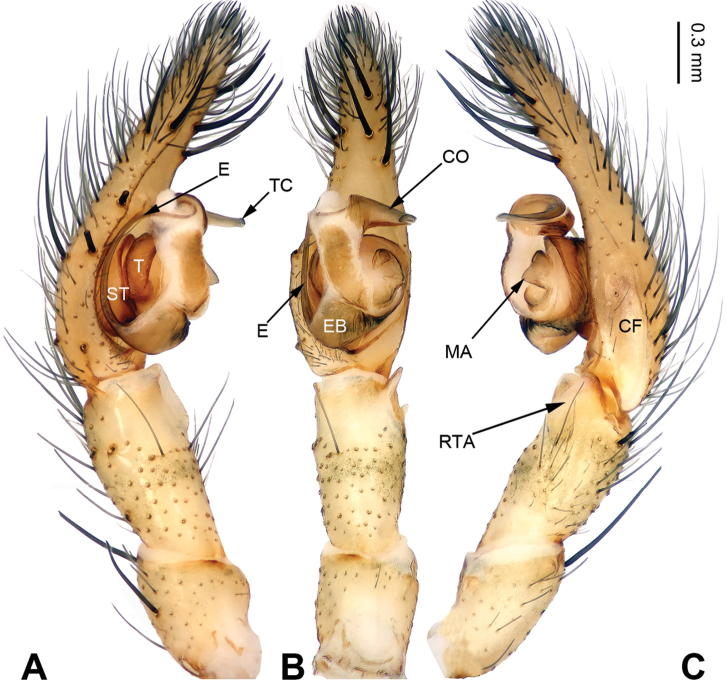
Left palp of *Pireneitega burqinensis* sp. n., male holotype. **A** Prolateral view **B** Ventral view **C** Retrolateral view. Scale bar: equal for **A, B, C**.

The chelicerae of all *Pireneitega* have 3 promarginal and 3 retromarginal teeth; other coelotines usually have 2 or 4 retromarginal teeth. The females of this genus can be separated from other coelotines by the widely separated long epigynal teeth, the large epigynal atrium with weakly sclerotized septum, and broad copulatory ducts (Fig. 6A–B); other coelotines usually have a small epigynal atrium, the short epigynal teeth and narrow copulatory ducts. The males of this genus can be distinguished from other coelotines by with an elongated and flattened conductor which is usually twisted into a circle horizontally or vertically and a large median apophysis (Fig. 1A–C); other coelotines usually have a broad or short conductor and a reduced or indistinct median apophysis.

##### Description.

Described in Wang (2002, sub *Paracoelotes*).

##### Composition.

Twenty-one *Pireneitega* species are known from Germany, Italy, Spain, France, Turkey, Georgia, Azerbaijan, Uzbekistan, Tajikistan, Russia, South Korea, Japan and China (World Spider Catalog 2016). Eleven *Pireneitega* species were known from China before the current study, including *Pireneitega involuta* (Wang et al., 1990) (♂♀), *Pireneitega liansui* (Bao & Yin, 2004) (♀), *Pireneitega luctuosa* (L. Koch, 1878) (♂♀), *Pireneitega luniformis* (Zhu & Wang, 1994) (♂♀), *Pireneitega neglecta* (Hu, 2001) (♀), *Pireneitega spinivulva* (Simon, 1880) (♂♀), *Pireneitega taishanensis* (Wang et al., 1990) (♂♀), *Pireneitega taiwanensis* Wang & Ono, 1998 (♂♀), *Pireneitega tianchiensis* (♂♀), *Pireneitega triglochinata* (Zhu & Wang, 1991) (♂♀), and *Pireneitega xinping* Zhang, Zhu & Song, 2002 (♂♀).

#### Pireneitega burqinensis

Taxon classificationAnimaliaAraneaeAgelenidae

Zhao & Li sp. n.

http://zoobank.org/403E6B13-C543-4EE1-9387-6CB2F9F9005A

Figs 1 , 2 , 17

##### Type material.

**Holotype** ♂: China: Xinjiang, Ili Kazakh Autonomous Prefecture, Altay Prefecture: Burqin County, on the way from Jiadenyu to Hemu Village, birch forest, N48°31'08", E87°11'13", 1469 m, 23.VII.2013, Z. Yao and Z. Zhao. **Paratype**: 1♀, same data as holotype.

##### Other material studied.

*Pireneitega tianchiensis*: 1♀1♂ (Figs 12–13): China: Xinjiang, Changji Hui Autonomous Prefecture: Fukang City, Crater Lake Scenic Spot (in Chinese: Tianchi), N43°54'05", E88°07'29", 1878 m, 16.VII.2013, Z. Yao and Z. Zhao.

##### Etymology.

The specific name refers to the type locality; adjective.

##### Diagnosis.

**Figure 2.**
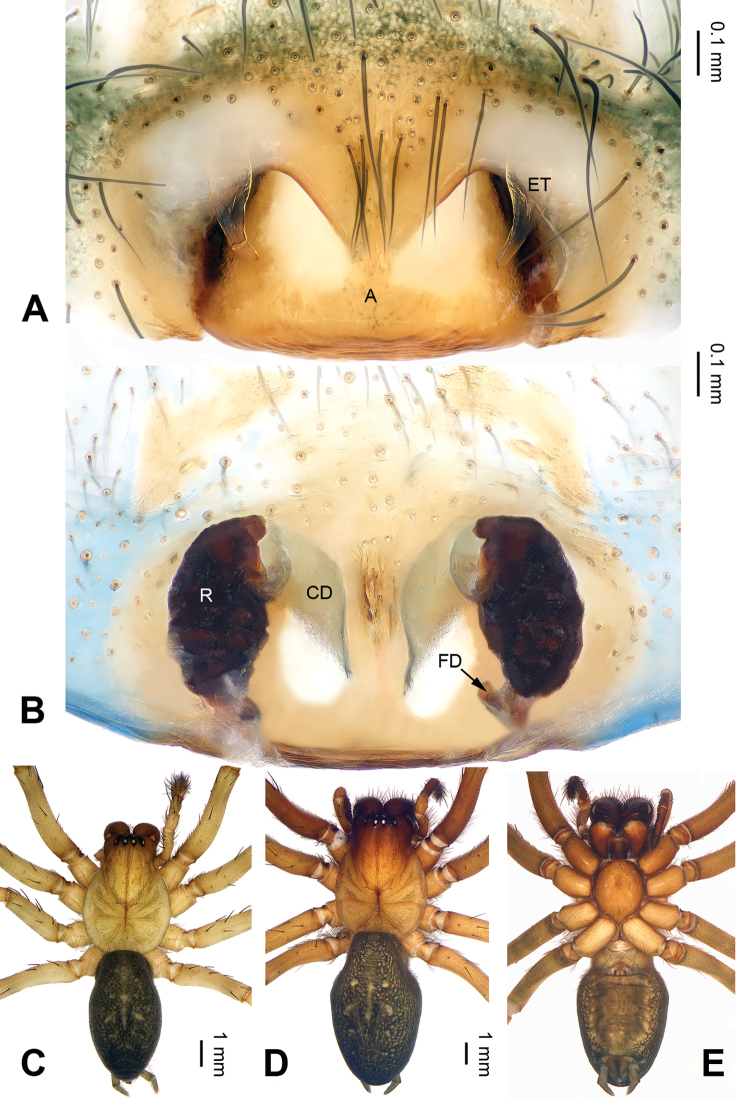
*Pireneitega burqinensis* sp. n., female paratype and male holotype. **A** Epigyne, ventral view **B** Vulva, dorsal view **C** Male habitus, dorsal view **D** Female habitus, dorsal view **E** Female habitus, ventral view. Scale bars: equal for **D, E**.

The male can be distinguished from all other *Pireneitega* species, except *Pireneitega tianchiensis*, by having narrow tibia and tarsus. From *Pireneitega tianchiensis*, it can be distinguished by the nearly trapezoidal embolic base (while *Pireneitega tianchiensis* has the nearly fingernail-shaped embolic base) (cf. Figs 1 and 12; Wang et al. 1990: figs 81–83). The female can be distinguished from all other *Pireneitega* species, except *Pireneitega tianchiensis*, by having the weakly sclerotized tip of septum and the closely spaced copulatory opening. From *Pireneitega tianchiensis*, it can be distinguished by the sharply narrowed epigynal teeth (while in *Pireneitega tianchiensis* the epigynal teeth are broad and nearly horn-shaped) (cf. Figs 2A–B and 13A–B; Wang et al. 1990: figs 84–85).

##### Description.

**Male (holotype)**: Total length 12.25. Carapace 5.25 long, 4.25 wide. Abdomen 7.00 long, 4.00 wide. Eye sizes and interdistances: AME 0.27, ALE 0.28, PME 0.23, PLE 0.23; AME-AME 0.10, AME-ALE 0.06, PME-PME 0.17, PME-PLE 0.23. Leg measurements: I: 18.40 (5.50, 6.25, 4.50, 2.15); II: 17.25 (5.00, 6.00, 4.25, 2.00); III: 16.15 (4.75, 5.15, 4.25, 2.00); IV: 20.15 (5.75, 6.50, 5.75, 2.15). Carapace greenish, the radial grooves indistinct, with black lateral margins. Abdomen blackish, with yellow spots. Palp: patellar apophysis absent; tibia short, about 1/2 length of cymbium; RTA short, about 1/3 of tibial length, without pointed tip, extended beyond the tibia; cymbial furrow short, about 1/3 length of cymbium; conductor long, nearly hook-shaped, with one loop; median apophysis broad and nearly triangular; embolus with broad and nearly trapezoidal base, beginning at 6:30 o’clock position (Fig. 1A–C).

**Female** (paratype): Total length 9.50. Carapace 4.50 long, 3.60 wide. Abdomen 5.00 long, 3.00 wide. Eye sizes and interdistances: AME 0.20, ALE 0.25, PME 0.18, PLE 0.18; AME-AME 0.10, AME-ALE 0.05, PME-PME 0.15, PME-PLE 0.23. Leg measurements: I: 17.90 (5.00, 6.00, 4.75, 2.15); II: 17.00 (5.00, 5.50, 4.50, 2.00); III: 16.00 (4.75, 5.00, 4.50, 1.75); IV: 19.75 (5.50, 6.00, 6.00, 2.25). Carapace reddish, with brown lateral margins. Abdomen blackish, with yellow sigilla. Epigyne: epigynal teeth light-colored and hyaline, about 0.5 times as long as epigynal atrium, located in anterior part of epigynal atrium; septum about 0.6 times as long as wide, nearly triangular; atrium about 1.2 times as long as wide, with weakly sclerotized posterior margin and nearly triangular, about two times as long as septum, subequal to the width of septum; receptacles about two times as long as wide, located in the posterior part of epigyne; copulatory opening indistinct; hoods indistinct (Fig. 2A–B).

##### Distribution.

Known only from the type locality (Fig. 17).

#### Pireneitega fuyunensis

Taxon classificationAnimaliaAraneaeAgelenidae

Zhao & Li sp. n.

http://zoobank.org/21C8277B-74D3-4C6D-9E4F-0F8C35E9DDD4

Figs 3 , 4 , 17

##### Type material.

**Holotype** ♂: China: Xinjiang, Ili Kazakh Autonomous Prefecture, Altay Prefecture: Fuyun County, Ocoa Sea Breeze, Erqis Grand Canyon, on the way from the Carla Chale Waterfall to the Shenzhong Mountain, N47°19'28", E90°01'51", 1355 m, 19.VII.2013, J. Liu, K. Meng, Z. Yao, and Z. Zhao. **Paratypes**: 4♀1♂, same data as holotype.

##### Etymology.

The specific name refers to the type locality; adjective.

##### Diagnosis.

**Figure 3.**
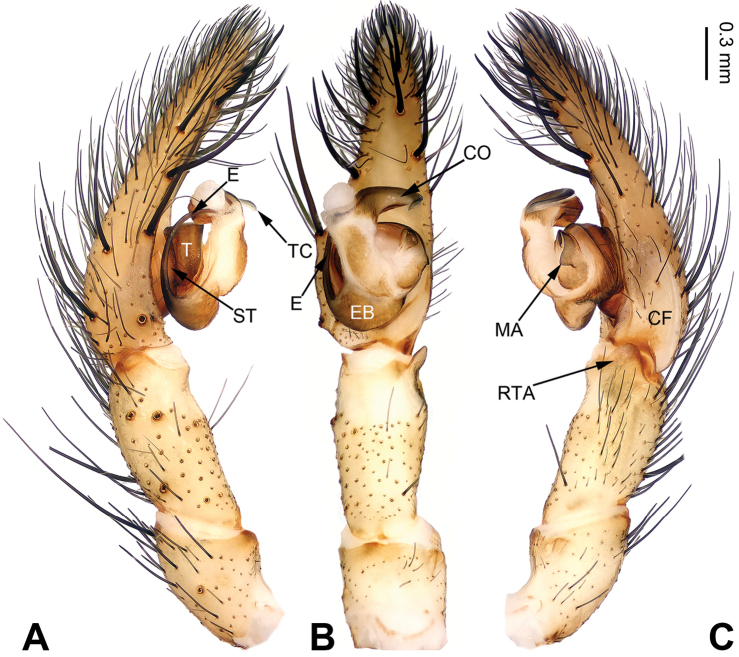
Left palp of *Pireneitega fuyunensis* sp. n., male holotype. **A** Prolateral view **B** Ventral view **C** Retrolateral view. Scale bar: equal for **A, B, C**.

**Figure 4.**
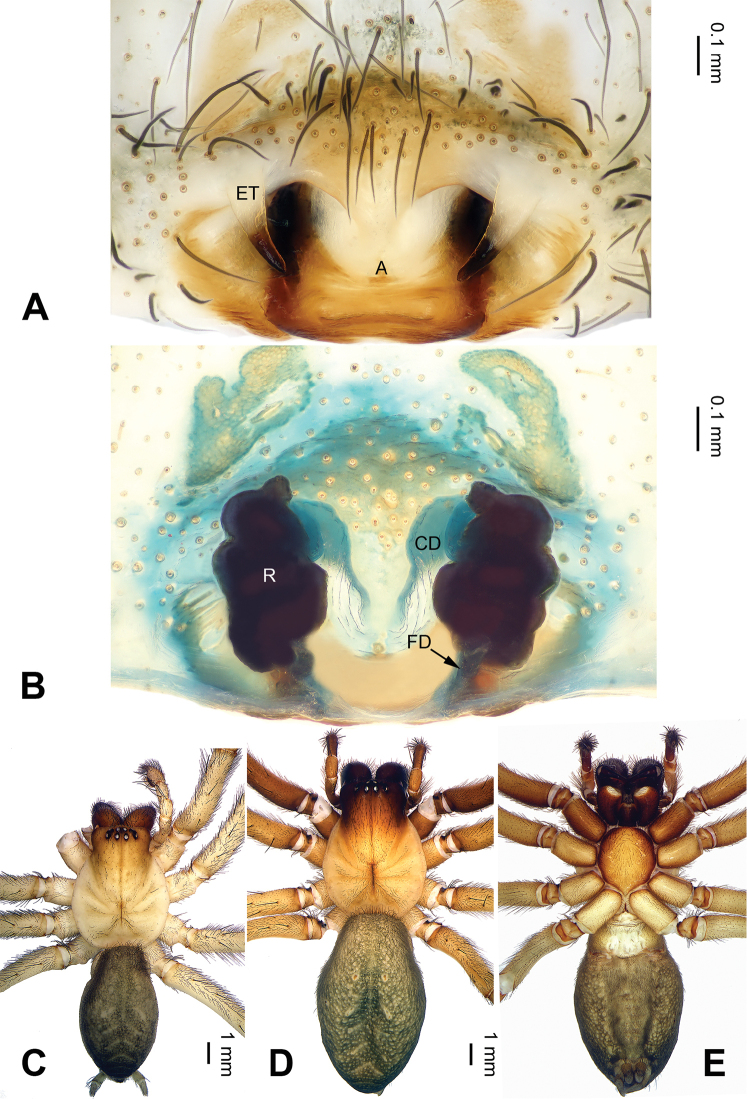
*Pireneitega fuyunensis* sp. n., female paratype and male holotype. **A** Epigyne, ventral view **B** Vulva, dorsal view **C** Male habitus, dorsal view **D** Female habitus, dorsal view **E** Female habitus, ventral view. Scale bars: equal for **D, E**.

The male can be distinguished from all other *Pireneitega* species, except *Pireneitega burqinensis* sp. n. and *Pireneitega tianchiensis*, by having a hook-shaped conductor, and can be distinguished from these two species by the small and narrow median apophysis (the broad and nearly fins-shaped apophysis in *Pireneitega burqinensis* sp. n. and *Pireneitega tianchiensis*) (cf. Figs 1, 3 and 12; Wang et al.1990: figs 81–83). The female can be distinguished from all other *Pireneitega* species, except *Pireneitega burqinensis* sp. n. and *Pireneitega tianchiensis*, by having short receptacles and the large epigynal atrium, and can be distinguished from these two species by the receptacles, about 1.5 times as long as wide (about two times longer than wide in *Pireneitega burqinensis* sp. n. and about 1.2 times in *Pireneitega tianchiensis*) (cf. Figs 2A–B, 4A–B and 13A–B; Wang et al. 1990: figs 84–85).

##### Description.

**Male (holotype)**: Total length 12.18. Carapace 5.45 long, 4.75 wide. Abdomen 6.73 long, 3.50 wide. Eye sizes and interdistances: AME 0.26, ALE 0.30, PME 0.25, PLE 0.25; AME-AME 0.08, AME-ALE 0.10, PME-PME 0.20, PME-PLE 0.20. Leg measurements: I: 22.63 (6.25, 7.69, 6.09, 2.60); II: 22.15 (6.20, 7.45, 6.00, 2.50); III: 20.75 (5.50, 7.00, 6.00, 2.25); IV: 25.67 (7.05, 8.33, 7.69, 2.60). Carapace beige, the radial grooves indistinct. Abdomen blackish, with gray herringbone pattern. Palp: patellar apophysis absent; tibia long, about 1/2 length of the cymbium; RTA short, about 1/4 of tibial length; cymbial furrow short, about 1/4 length of cymbium; conductor short, with tapering apex, with one loop; embolus with nearly tongue-shaped base, beginning at 7:00 o’clock position (Fig. 3A–C).

**Female** (one of paratypes): Total length 13.80. Carapace 5.25 long, 4.50 wide. Abdomen 8.55 long, 4.75 wide. Eye sizes and interdistances: AME 0.25, ALE 0.30, PME 0.24, PLE 0.24; AME-AME 0.10, AME-ALE0.10, PME-PME 0.20, PME-PLE 0.28. Leg measurements: I: 18.75 (5.50, 6.50, 4.50, 2.25); II: 18.00 (5.25, 6.25, 4.50, 2.00); III: 16.98 (4.99, 5.74, 4.50, 1.75); IV: 21.60 (6.10, 7.25, 6.03, 2.22). Carapace yellowish, with brown lateral margins. Abdomen khaki, with yellow sigilla and herringbone pattern. Epigyne: epigynal teeth long and thin, about 0.8 times as long as epigynal atrium; septum about 0.5 times as long as wide, with the weakly sclerotized tip, nearly triangular; epigynal atrium about 1.5 times as long as wide, with well delimited posterior margin, about two times as long as septum, about 0.7 times as wide as septum; receptacles about 1.5 times as long as wide; copulatory opening distinct; hoods indistinct (Fig. 4A–B).

##### Distribution.

Known only from the type locality (Fig. 17).

#### Pireneitega gongliuensis

Taxon classificationAnimaliaAraneaeAgelenidae

Zhao & Li sp. n.

http://zoobank.org/31392283-8A5E-4011-9EEA-D84A3CB4D54B

Figs 5 , 6 , 17

##### Type material.

**Holotype** ♂: China: Xinjiang, Ili Kazakh Autonomous Prefecture: Gongliu County, N43°22'23", E81°51'45", 1515 m, 9.VIII.2013, J. Liu, K. Meng, Z. Yao, and Z. Zhao. **Paratypes**: 2♀2♂, same data as holotype.

##### Etymology.

The specific name refers to the type locality; adjective.

##### Diagnosis.

**Figure 5.**
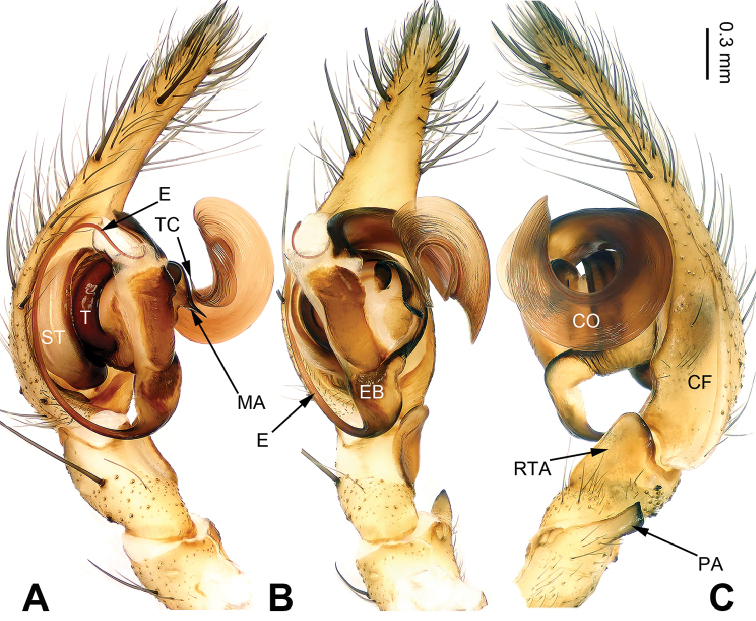
Left palp of *Pireneitega gongliuensis* sp. n., male holotype. **A** Prolateral view **B** Ventral view **C** Retrolateral view. Scale bar: equal for **A, B, C**.

**Figure 6.**
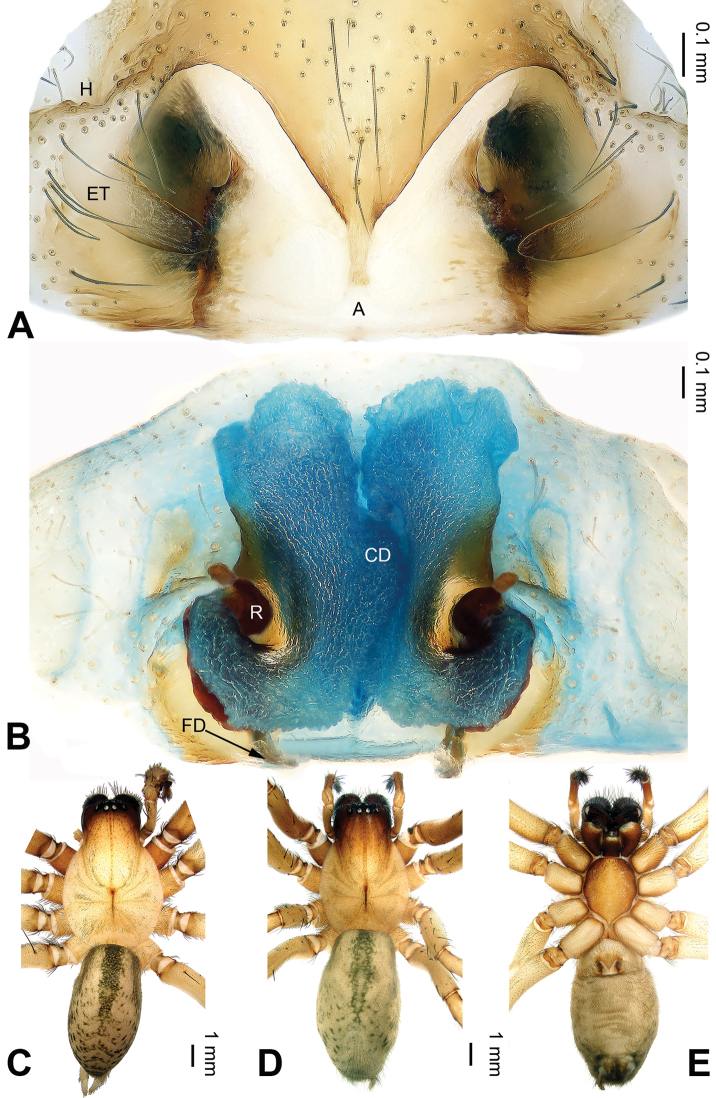
*Pireneitega gongliuensis* sp. n., female paratype and male holotype. **A** Epigyne, ventral view **B** Vulva, dorsal view **C** Male habitus, dorsal view **D** Female habitus, dorsal view **E** Female habitus, ventral view. Scale bars: equal for **D, E**.

The male can be distinguished from all other *Pireneitega* species, except *Pireneitega involuta* and *Pireneitega xinping*, by having a broad conductor and thick patellar apophysis, and can be distinguished from these two species by the tapering tip of conductor (the rounded tip of conductor in *Pireneitega involuta* and *Pireneitega xinping*) (cf. Fig. 5; Wang et al. 1990: figs 13–15; Zhang et al. 2002: figs 9–10). The female can be distinguished from all other *Pireneitega* species, except for *Pireneitega xinping*, by having large copulatory ducts, and can be distinguished from this species by the short and thick epigynal teeth, about 0.5 times as long as epigynal atrium (the long and narrow epigynal teeth in *Pireneitega xinping*, subequal to the length of epigynal atrium) (cf. Fig. 6A–B; Zhang et al. 2002: figs 7–8).

##### Description.

**Male (holotype)**: Total length 13.27. Carapace 6.09 long, 4.49 wide. Abdomen 7.18 long, 3.21 wide. Eye sizes and interdistances: AME 0.20, ALE 0.20, PME 0.23, PLE 0.22; AME-AME 0.08, AME-ALE 0.08, PME-PME 0.13, PME-PLE 0.25. Leg measurements: I: 19.87 (5.14, 6.73, 5.00, 3.00); II: 18.50 (5.00, 6.00, 4.75, 2.75); III: 16.70 (4.60, 5.10, 4.50, 2.50); IV: 21.59 (5.45, 6.73, 6.41, 3.00). Carapace yellow. Abdomen gray, with nearly chevrons-shaped stripes. Palp: patellar apophysis long, more than half of the tibia; tibia short, about 1/4 of tarsus; RTA subequal to the tibial length; cymbial furrow long, more than half of cymbium; conductor broad and long, with two loops; embolus with broad base, beginning at 5:30 o’clock position (Fig. 5A–C).

**Female** (one of paratypes): Total length 12.18. Carapace 5.13 long, 3.80 wide. Abdomen 7.05 long, 3.50 wide. Eye sizes and interdistances: AME 0.20, ALE 0.25, PME 0.21, PLE 0.22; AME-AME 0.10, AME-ALE0.07, PME-PME 0.15, PME-PLE 0.20. Leg measurements: I: 15.28 (4.50, 5.26, 3.40, 2.12); II: 14.91 (4.40, 5.13, 3.33, 2.05); III: 12.99 (3.72, 4.17, 3.30, 1.80); IV: 17.96 (4.75, 5.96, 5.00, 2.25). Carapace yellowish. Abdomen gray, with green spots. Epigyne: epigynal teeth about 0.5 times as long as atrium, light-colored; septum about 0.5 times as long as wide; atrium large, the length subequal to the width, with weakly sclerotized posterior margin, about 1.3 times as long as septum, about 0.6 times as long as septum; receptacles about 1.5 times as long as wide, almost covered by copulatory ducts; copulatory opening distinct; hoods distinct (Fig. 6A–B).

##### Distribution.

Known only from the type locality (Fig. 17).

#### Pireneitega huochengensis

Taxon classificationAnimaliaAraneaeAgelenidae

Zhao & Li sp. n.

http://zoobank.org/C537A7D3-8C52-4740-A699-0C104A51640C

Figs 7 , 8 , 17

##### Type material.

**Holotype** ♂: China: Xinjiang, Ili Kazakh Autonomous Prefecture: Huocheng County, Sarbulak town, N44°13'14", E81°10'13", 987 m, 4.VIII.2013, J. Liu, K. Meng, Z. Yao, and Z. Zhao. **Paratypes**: 3♀3♂, same data as holotype.

##### Etymology.

The specific name refers to the type locality; adjective.

##### Diagnosis.

**Figure 7.**
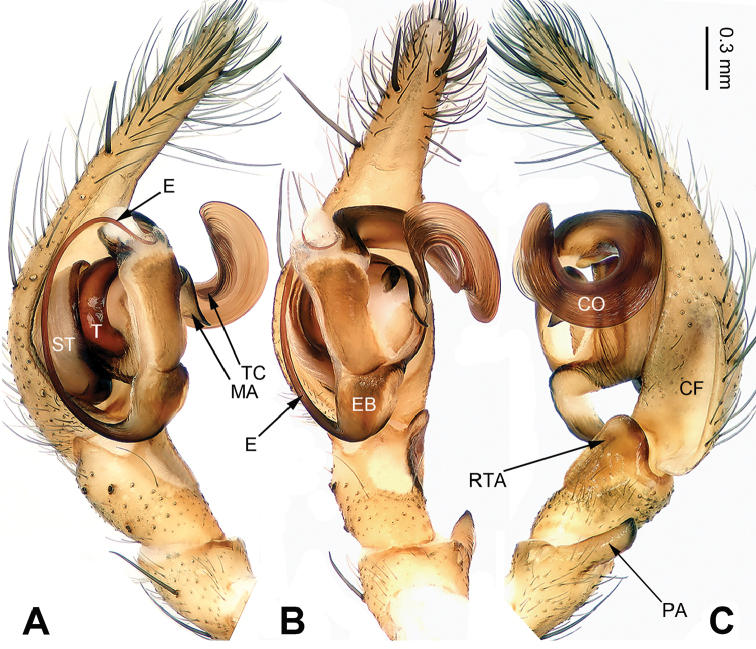
Left palp of *Pireneitega huochengensis* sp. n., male holotype. **A** Prolateral view **B** Ventral view **C** Retrolateral view. Scale bar: equal for **A, B, C**.

**Figure 8.**
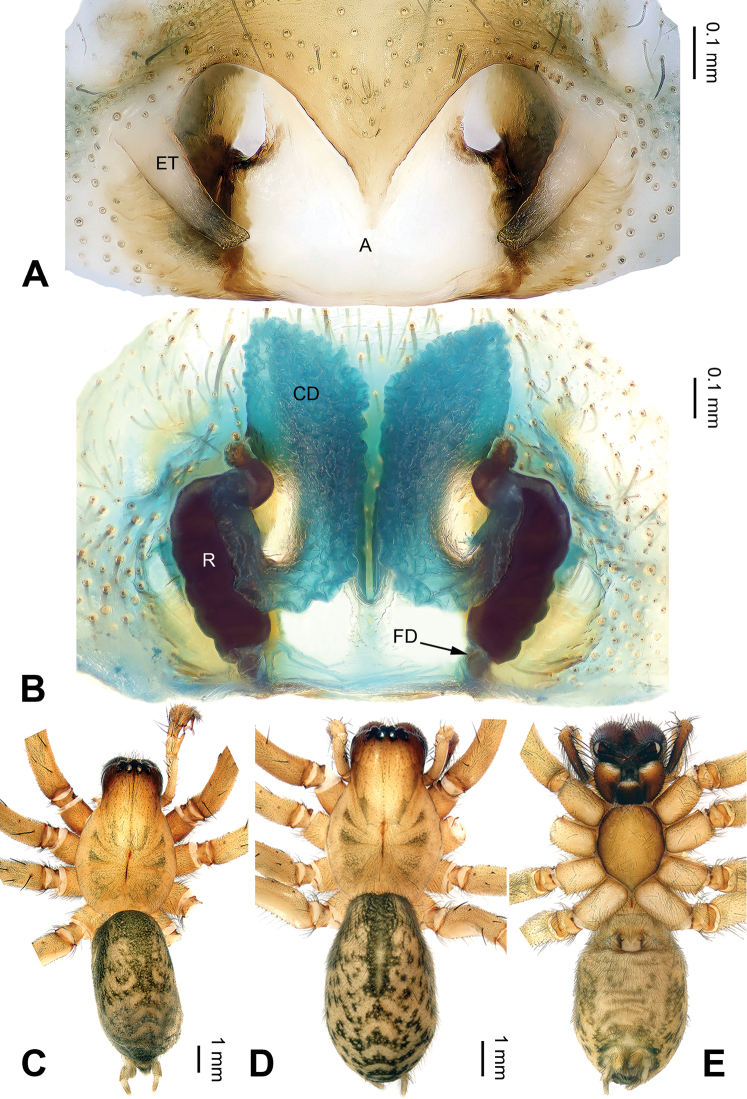
*Pireneitega huochengensis* sp. n., female paratype and male holotype. **A** Epigyne, ventral view **B** Vulva, dorsal view **C** Male habitus, dorsal view **D** Female habitus, dorsal view **E** Female habitus, ventral view. Scale bars: equal for **D, E**.

The male can be distinguished from all other *Pireneitega* species, except *Pireneitega gongliuensis* sp. n., *Pireneitega involuta* and *Pireneitega xinping*, by having a broad conductor and thick patellar apophysis, and can be distinguished from these species by the blunt tip of patellar apophysis (the tapering tip in *Pireneitega involuta* and *Pireneitega xinping*, the nearly rectangular in *Pireneitega gongliuensis* sp. n.) (cf. Figs 5 and 7; Wang et al. 1990: figs 13–15; Zhang et al. 2002: figs 9–10). The female can be distinguished from all other *Pireneitega* species, except for *Pireneitega gongliuensis* sp. n., by having long and broad epigynal teeth, and can be distinguished from this species by the blunt tip of epigynal teeth (the tapering tip in *Pireneitega gongliuensis* sp. n.) (cf. Figs 6A–B and 8A–B).

##### Description.

**Male (holotype)**: Total length 11.54. Carapace 5.13 long, 3.60 wide. Abdomen 6.41 long, 3.00 wide. Eye sizes and interdistances: AME 0.20, ALE 0.24, PME 0.24, PLE 0.24; AME-AME 0.12, AME-ALE 0.06, PME-PME 0.14, PME-PLE 0.16. Leg measurements: I: 15.15 (4.00, 5.20, 3.75, 2.20); II: 14.15 (3.75, 4.75, 3.65, 2.00); III: 13.30 (3.50, 4.25, 3.75, 1.80); IV: 17.50 (4.75, 5.40, 5.10, 2.25). Carapace yellow. Abdomen black, with 2 types of spots, one group yellow, and another transversal spots. Palp: patellar apophysis long, subequal to the tibial length; tibia short, about 1/4 of the cymbium length; RTA long, subequal to the tibial length; cymbial furrow short, about 1/3 length of cymbium; conductor broad, with broad apex, with two loops; embolus with broad base, beginning at 6:00 o’clock position (Fig. 7A–C).

**Female** (one of paratypes): Total length 9.94. Carapace 4.49 long, 3.05 wide. Abdomen 5.45 long, 2.90 wide. Eye sizes and interdistances: AME 0.18, ALE 0.23, PME 0.24, PLE 0.30; AME-AME 0.10, AME-ALE 0.05, PME-PME 0.15, PME-PLE 0.10. Leg measurements: I: 11.25 (3.25, 4.00, 2.50, 1.50); II: 10.30 (3.00, 3.50, 2.50, 1.30); III: 9.70 (2.75, 3.00, 2.65, 1.30); IV: 13.75 (3.75, 4.25, 4.00, 1.75). Carapace yellow, with brown lateral margins. Abdomen yellow, with black and nearly chevrons-shaped stripes. Epigyne: epigynal teeth thick, about 0.7 times as long as epigynal atrium; septum with the well delimited tip, about 0.3 times as long as wide; atrium with well delimited posterior margin, the length subequal to the width, about 2.3 times as long as septum, about 0.8 times as wide as septum; receptacles long, about 2.7 times as long as wide; copulatory opening distinct; hoods indistinct (Fig. 8A–B).

##### Distribution.

Known only from the type locality (Fig. 17).

#### Pireneitega lini

Taxon classificationAnimaliaAraneaeAgelenidae

Zhao & Li sp. n.

http://zoobank.org/8269EC62-A11C-4CAC-A2E9-A8D1CAC6B1A3

Figs 9 , 17

##### Type material.

**Holotype** ♀: China: Xinjiang, Kizilsu Kirghiz Autonomous Prefecture: Akto County, N38°57'31", E75°30'16", 1833 m, 2.VIII.2014, Y. Lin.

##### Etymology.

The specific name is a patronym in honor of the collector Yucheng Lin; noun (name) in genitive case.

##### Diagnosis.

**Figure 9.**
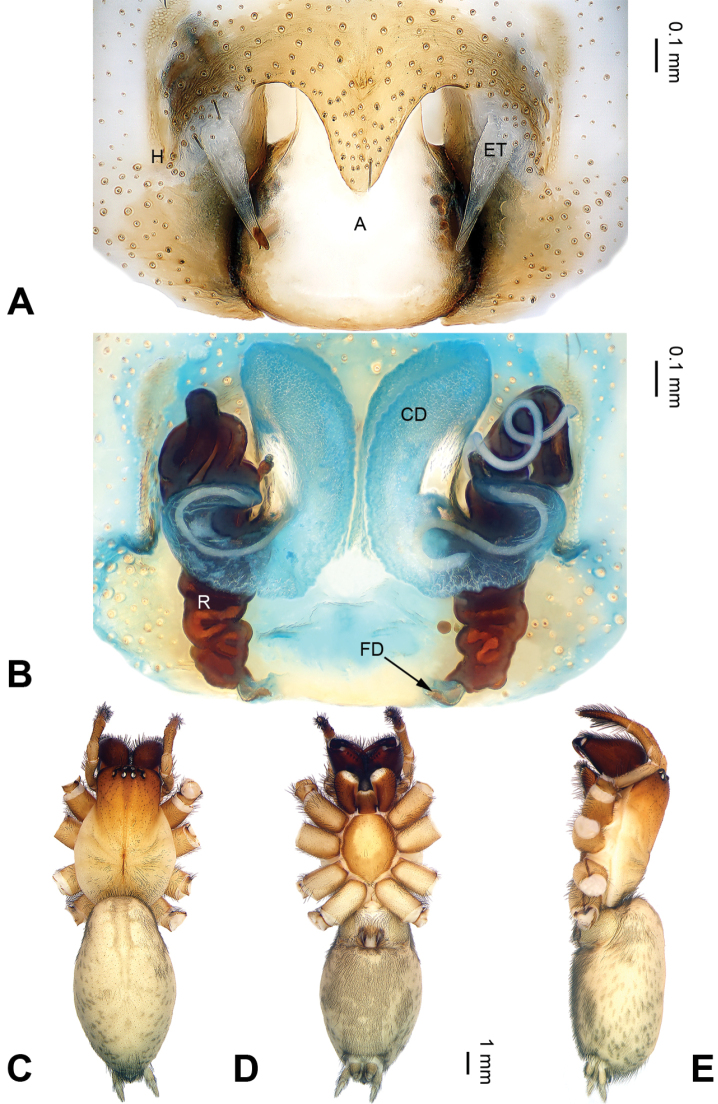
*Pireneitega lini* sp. n., female holotype. **A** Epigyne, ventral view **B** Vulva, dorsal view **C** Female habitus, dorsal view **D** Female habitus, dorsal view **E** Female habitus, ventral view. Scale bars: equal for **C, D, E**.

The female can be distinguished from all other *Pireneitega* species, except for *Pireneitega luniformis*, by having spiral receptacles, and can be distinguished from this species by the narrow and straight epigynal teeth (the broad and bent epigynal teeth in *Pireneitega luniformis*) (cf. Fig. 9A–B; Zhu and Wang 1994: figs 5–6).

##### Description.

**Female (holotype)**: Total length 13.82. Carapace 5.49 long, 4.50 wide. Abdomen 8.33 long, 4.50 wide. Eye sizes and interdistances: AME 0.25, ALE 0.30, PME 0.25, PLE 0.24; AME-AME 0.10, AME-ALE 0.08, PME-PME 0.19, PME-PLE 0.25. Leg measurements: I: 18.14 (5.00, 6.41, 4.17 2.56); II: 16.99 (4.81, 5.77, 4.17, 2.24); III: 16.69 (4.49, 5.45, 4.50, 2.25); IV: 21.68 (5.78, 6.73, 6.41, 2.76). Carapace yellow. Abdomen beige, with gray spots. Epigyne: epigynal teeth long and narrow, about 0.7 times as long as epigynal atrium; septum with the well delimited tip, about 0.6 times as long as wide; epigynal atrium with well delimited posterior margin, about two times as long as wide, about 2.5 times as long as septum, about 0.7 times as wide as septum; receptacles long, about three times as long as wide, mightily spiral; copulatory opening distinct; hoods distinct (Fig. 9A–B).

##### Distribution.

Known only from the type locality (Fig. 17).

#### Pireneitega liui

Taxon classificationAnimaliaAraneaeAgelenidae

Zhao & Li sp. n.

http://zoobank.org/A633D7BA-526B-4393-BFD9-DDFE370EC052

Figs 10 , 11 , 17

##### Type material.

**Holotype** ♂: China: Xinjiang, Ili Kazakh Autonomous Prefecture: Xinyuan County, N43°21'58", E84°21'34", 2010 m, 12.VIII.2013, J. Liu. **Paratype**: 1♀, same data as holotype.

##### Etymology.

The specific name is a patronym in honor of the collector Jincheng Liu; noun (name) in genitive case.

##### Diagnosis.

**Figure 10.**
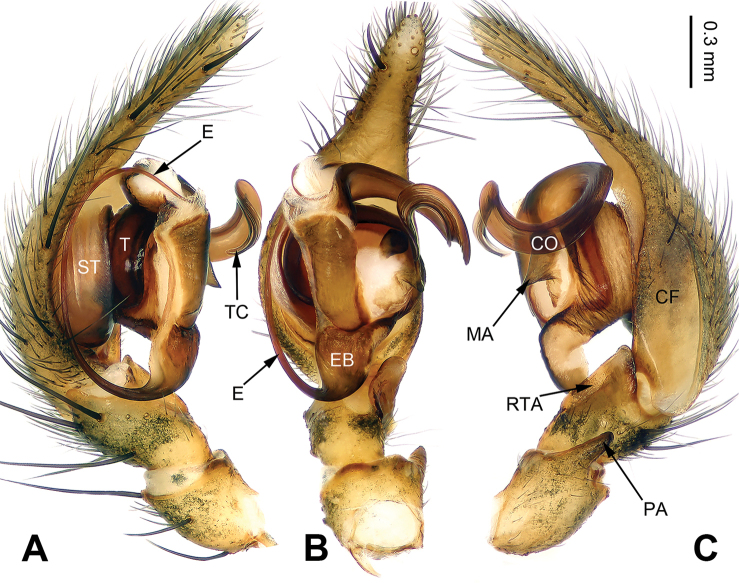
Left palp of *Pireneitega liui* sp. n., male holotype. **A** Prolateral view **B** Ventral view **C** Retrolateral view. Scale bar: equal for **A, B, C**.

**Figure 11.**
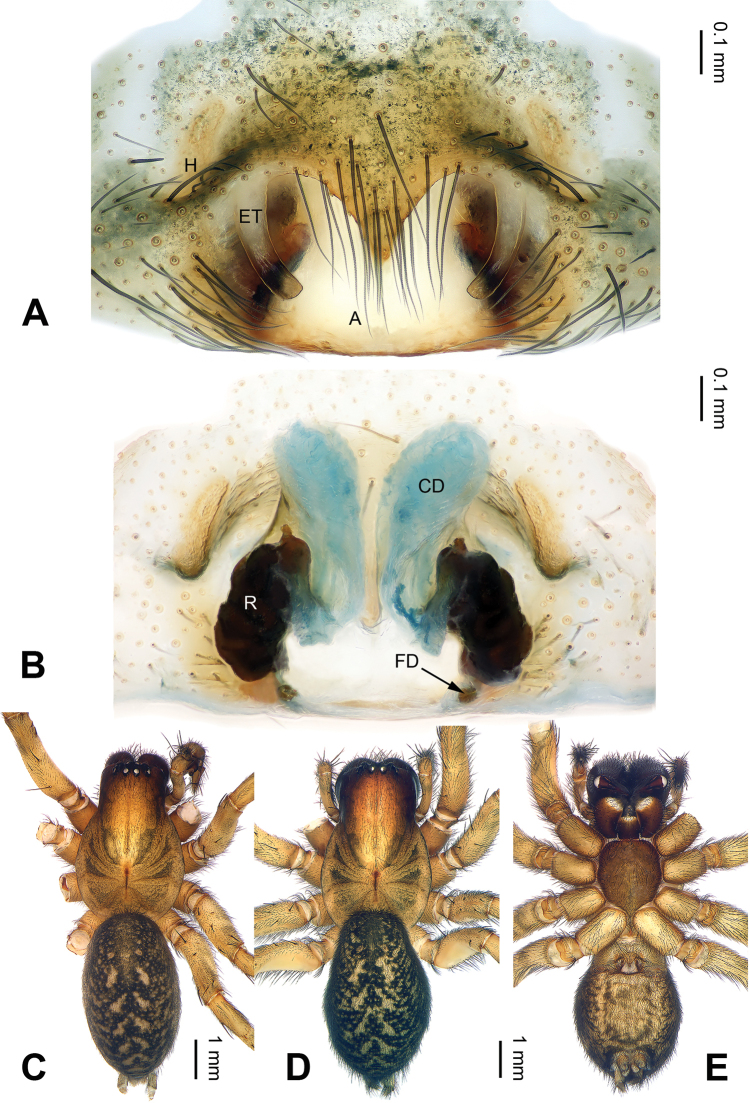
*Pireneitega liui* sp. n., female paratype and male holotype. **A** Epigyne, ventral view **B** Vulva, dorsal view **C** Male habitus, dorsal view **D** Female habitus, dorsal view **E** Female habitus, ventral view. Scale bars: equal for **D, E**.

The male can be easily distinguished from all the other *Pireneitega* species, except *Pireneitega luniformis*, by having a long and narrow conductor, and can be distinguished from this species by the blunt tip of the patellar apophysis (the tapering tip of conductor and the patellar apophysis in *Pireneitega luniformis*) (cf. Fig. 10; Zhu and Wang 1994: figs 7–8). The female can be distinguished from all other *Pireneitega* species, except *Pireneitega major* (Kroneberg, 1875) by having the nearly trapezoidal epigynal atrium, and can be distinguished from this species by the abrupt tip of epigynal teeth (the pointed tip of teeth in *Pireneitega major*) (cf. Fig. 11A–B; Kroneberg 1875: fig. 6).

##### Description.

**Male (holotype)**: Total length 8.25. Carapace 3.75 long, 2.85 wide. Abdomen 4.50 long, 2.50 wide. Eye sizes and interdistances: AME 0.18, ALE 0.25, PME 0.18, PLE 0.18; AME-AME 0.08, AME-ALE 0.05, PME-PME 0.10, PME-PLE 0.18. Leg measurements: I: 12.55 (3.50, 4.25, 2.80, 2.00); II: 11.75 (3.25, 3.75, 3.00, 1.75); III: 11.00 (3.00, 3.50, 3.00, 1.50); IV: 15.00 (4.00, 4.50, 4.50, 2.00). Carapace yellow, the radial grooves indistinct, with black lateral margins. Abdomen brown, with yellow herringbone pattern. Palp: patellar apophysis thick, with the blunt tip; RTA long, subequal to the length of tibia, without pointed tip; cymbial furrow short, about 1/3 length of cymbium; conductor broad and curving, with blunt apex; median apophysis small, with pointed tip; embolus with broad base, beginning at 6:00 o’clock position (Fig. 10A–C).

**Female** (paratype): Total length 8.75. Carapace 4.00 long, 3.25 wide. Abdomen 4.75 long, 3.00 wide. Eye sizes and interdistances: AME 0.18, ALE 0.25, PME 0.20, PLE 0.20; AME-AME 0.05, AME-ALE 0.08, PME-PME 0.15, PME-PLE 0.23. Leg measurements: I: 11.40 (3.25, 4.00, 2.65, 1.50); II: 10.90 (3.25, 3.65, 2.50, 1.50); III: 10.25 (3.00, 3.25, 2.50, 1.50); IV: 13.00 (3.75, 4.25, 3.50, 1.50). Carapace yellow, with black lateral margins. Abdomen blackish, with yellow herringbone pattern. Epigyne: epigynal teeth broad and blunt, about 0.5 times as long as atrium; septum with the well delimited tip, about 0.5 times as long as wide; atrium with well delimited posterior margin, about 0.7 times as long as wide, about two times as long as septum, about 0.7 times as wide as septum; receptacles long, about 1.7 times as long as wide; copulatory opening distinct; hoods distinct (Fig. 11A–B).

##### Distribution.

Known only from the type locality (Fig. 17).

#### Pireneitega wensuensis

Taxon classificationAnimaliaAraneaeAgelenidae

Zhao & Li sp. n.

http://zoobank.org/0CFFC8E7-622A-4D9B-9F05-21D001F4B1AC

Figs 14 , 17

##### Type material.

**Holotype** ♂: China: Xinjiang, Aksu Prefecture, Wensu County, Bozidun Kirgiz Village, N41°44'37", E80°43'05", 1991 m, 22.VII.2014, J. Wu.

##### Etymology.

The specific name refers to the type locality; adjective.

##### Diagnosis.

**Figure 12.**
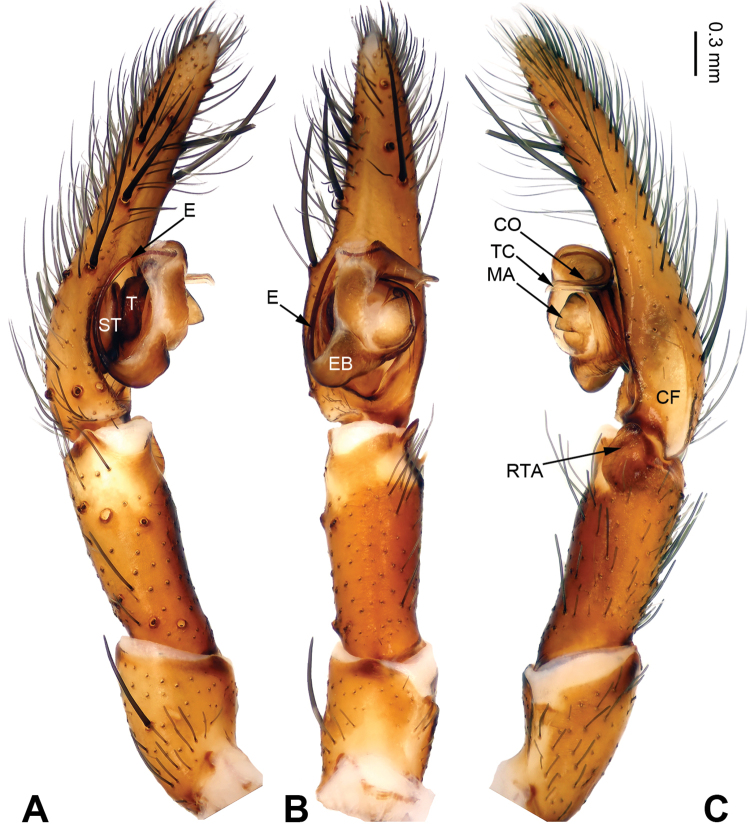
Left palp of *Pireneitega tianchiensis*, male from Crater Lake. **A** Prolateral view **B** Ventral view **C** Retrolateral view. Scale bar: equal for **A, B, C**.

The male can be distinguished from all other *Pireneitega* species, except *Pireneitega burqinensis* sp. n., *Pireneitega fuyunensis* sp. n. and *Pireneitega tianchiensis*, by having a hook-shaped conductor and the small bulb, and can be distinguished from these species by the long tibia, subequal to the length of cymbium (the short tibia, about 1/2 length of cymbium, in *Pireneitega burqinensis* sp. n., *Pireneitega fuyunensis* sp. n. and *Pireneitega tianchiensis*) (cf. Figs 1, 3, 12 and 14; Wang et al. 1990: figs 81–83).

**Figure 13.**
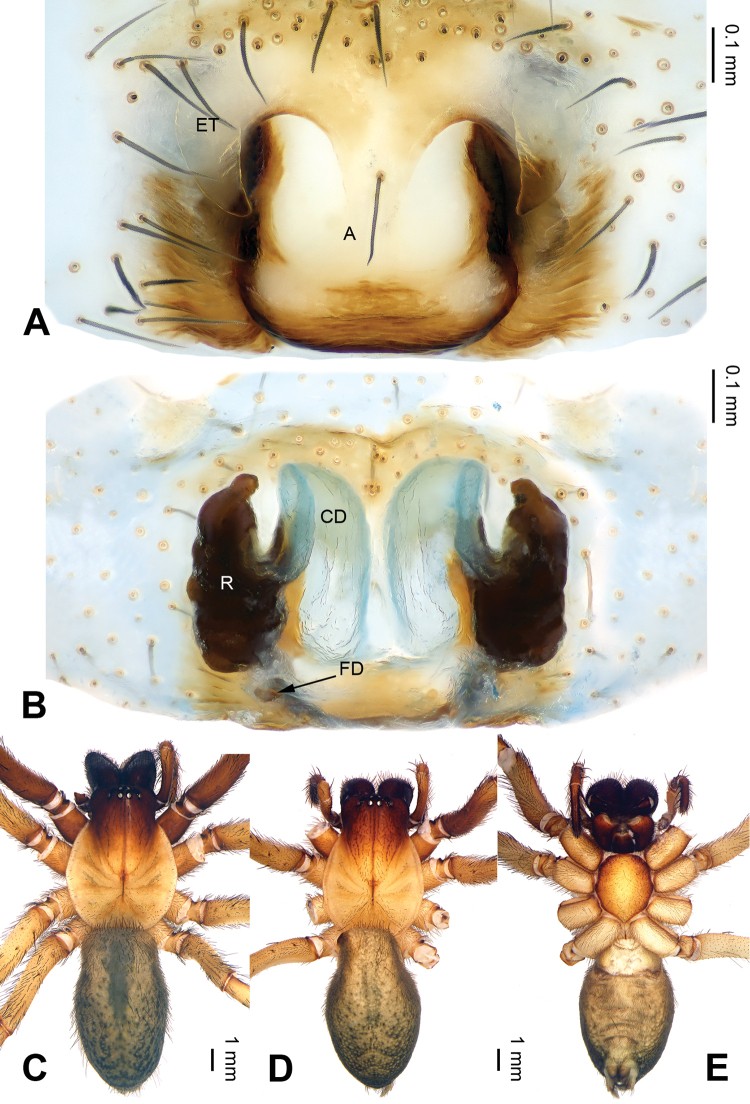
*Pireneitega tianchiensis*, specimens from Crater Lake. **A** Epigyne, ventral view **B** Vulva, dorsal view **C** Male habitus, dorsal view **D** Female habitus, dorsal view **E** Female habitus, ventral view. Scale bars: equal for **D, E**.

**Figure 14.**
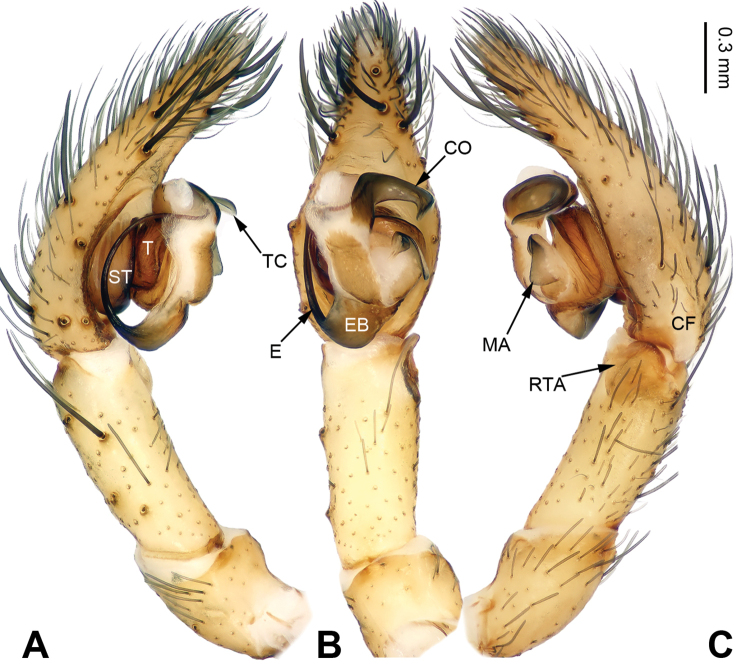
Left palp of *Pireneitega wensuensis* sp. n., male holotype. **A** Prolateral view **B** Ventral view **C** Retrolateral view. Scale bar: equal for **A, B, C**.

##### Description.

**Male (holotype)**: Total length 11.75. Carapace 4.50 long, 3.75 wide. Abdomen 7.25 long, 4.25 wide. Eye sizes and interdistances: AME 0.20, ALE 0.30, PME 0.25, PLE 0.25; AME-AME 0.05, AME-ALE 0.05, PME-PME 0.13, PME-PLE 0.13. Leg measurements: I: 16.75 (5.00, 5.75, 3.75, 2.25); II: 15.75 (4.50, 5.50, 3.75, 2.00); III: 15.25 (4.50, 5.00, 3.75, 2.00); IV: 19.75 (5.50, 6.25, 5.50, 2.50). Carapace yellow, the radial grooves indistinct, with black lateral margins. Abdomen blackish, with yellow spots. Palp: patellar apophysis absent; palpal tibia long; RTA short, about 1/4 of tibial length; cymbial furrow short, about 1/5 length of cymbium; conductor short, with blunt apex; median apophysis broad and nearly the flipper-shaped; embolus with broad and nearly square base, beginning at 6:30 o’clock position (Fig. 14A–C).

##### Distribution.

Known only from the type locality (Fig. 17).

#### Pireneitega wui

Taxon classificationAnimaliaAraneaeAgelenidae

Zhao & Li sp. n.

http://zoobank.org/D015D5F3-5D22-4545-8C81-FFFA6FE956FC

Figs 15 , 17

##### Type material.

**Holotype** ♂: China: Xinjiang, Kizilsu Kyrgyz Autonomous Prefecture, Akqi County, N40°47'32", E78°15'48", 3020 m, 25.VII.2014, J. Wu.

##### Etymology.

The specific name is a patronym in honor of the collector Jianglang Wu; noun (name) in genitive case.

##### Diagnosis.

**Figure 15.**
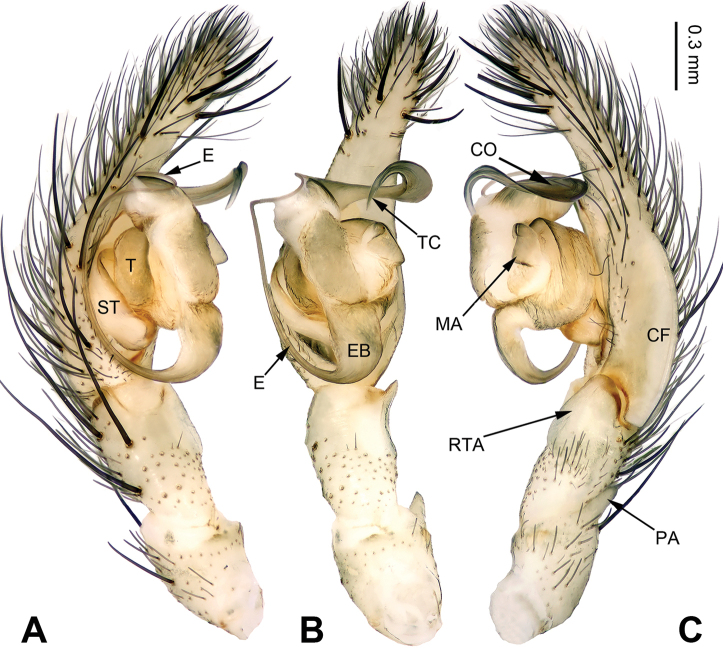
Left palp of *Pireneitega wui* sp. n., male holotype. **A** Prolateral view **B** Ventral view **C** Retrolateral view. Scale bar: equal for **A, B, C**.

The male can be distinguished from all other *Pireneitega* species, except *Pireneitega armeniaca* by having bended and narrow conductor, and can be distinguished from this species by the blunt tip of median apophysis (the tapering tip of median apophysis in *Pireneitega armeniaca*) (cf. Fig. 15; Brignoli 1978: figs 117–121).

##### Description.

**Male (holotype)**: Total length 8.98. Carapace 3.85 long, 2.75 wide. Abdomen 5.13 long, 3.00 wide. Eye sizes and interdistances: AME 0.18, ALE 0.23, PME 0.20, PLE 0.20; AME-AME 0.05, AME-ALE 0.05, PME-PME 0.10, PME-PLE 0.10. Leg measurements: I: 14.75 (4.25, 5.25, 3.25, 2.00); II: 13.00 (3.50, 4.50, 3.00, 2.00); III: 12.25 (3.50, 4.00, 3.25, 1.50); IV: 17.50 (5.00, 5.25, 5.00, 2.25). Carapace yellow. Abdomen beige, with gray spots. Palp: patellar apophysis thick; palpal tibia short, about 1/3 of cymbial length; RTA short, about 1/3 of tibial length; cymbial furrow short, about 1/3 length of cymbium; conductor narrow, with the tapering tip; median apophysis broad and nearly triangular; embolus with broad and nearly trapezoidal base, beginning at 7:00 o’clock position (Fig. 15A–C).

##### Distribution.

Known only from the type locality (Fig. 17).

#### Pireneitega yaoi

Taxon classificationAnimaliaAraneaeAgelenidae

Zhao & Li sp. n.

http://zoobank.org/9DFF584F-38EA-4FC3-AED2-176969E0D309

Figs 16 , 17

##### Type material.

**Holotype** ♀: China: Xinjiang, Ili Kazakh Autonomous Prefecture: Tacheng (Tarbagatay) Prefecture, Hoboksar Mongol Autonomous County, Songshugou, N46°59'21", E85°57'20", 1858 m, 29.VII.2013, Z. Yao and Z. Zhao. **Paratype**: 1♀, same data as holotype.

##### Etymology.

The specific name is a patronym in honor of the collector Zhiyuan Yao; noun (name) in genitive case.

##### Diagnosis.

**Figure 16.**
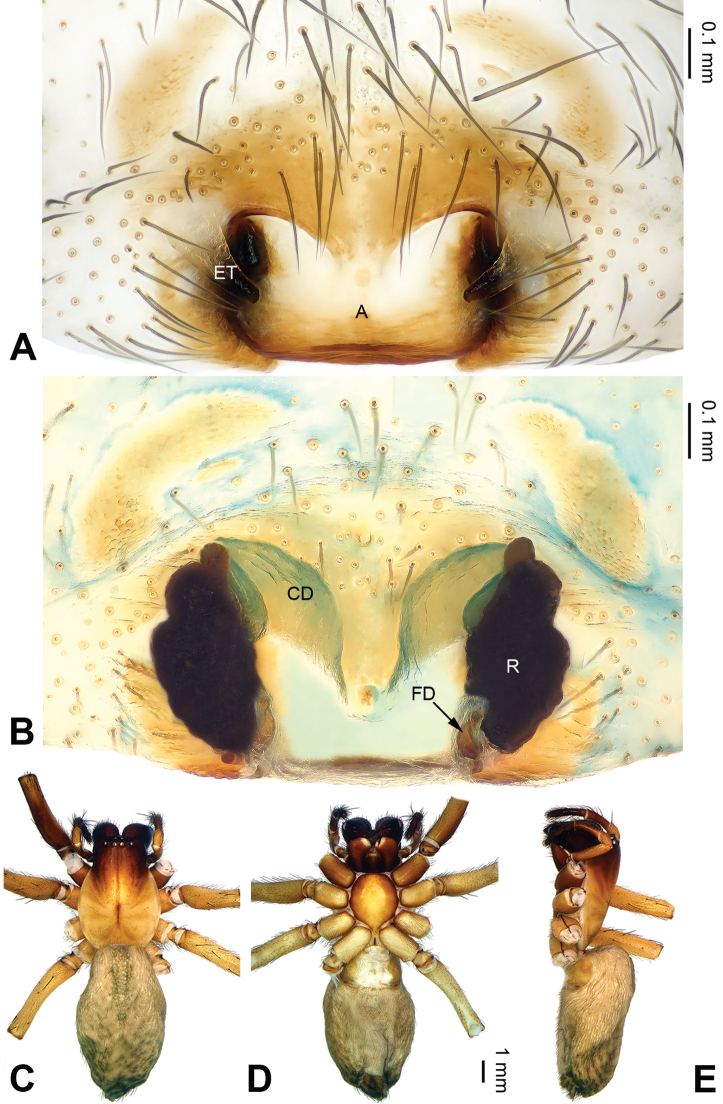
*Pireneitega yaoi* sp. n., female holotype. **A** Epigyne, ventral view **B** Vulva, dorsal view **C** Female habitus, dorsal view **D** Female habitus, dorsal view **E** Female habitus, ventral view. Scale bars: equal for **C, D, E**.

The female can be distinguished from all other *Pireneitega* species, except *Pireneitega burqinensis* sp. n., *Pireneitega fuyunensis* sp. n. and *Pireneitega tianchiensis*, by having the weakly sclerotized tip of septum. It can be distinguished from these three species by the nearly rectangular epigynal atrium (while *Pireneitega burqinensis* sp. n. has a nearly triangular atrium; and *Pireneitega tianchiensis* and *Pireneitega fuyunensis* sp. n., large and nearly square-shaped atrium) (cf. Figs 2A–B, 4A–B, 6A–B and 16A–B; Wang et al. 1990: figs 84–85).

##### Description.

**Female (holotype)**: Total length 12.25. Carapace 5.00 long, 4.25 wide. Abdomen 7.25 long, 4.50 wide. Eye sizes and interdistances: AME 0.23, ALE 0.27, PME 0.23, PLE 0.25; AME-AME 0.12, AME-ALE 0.08, PME-PME 0.13, PME-PLE 0.25. Leg measurements: I: 16.05 (4.90, 5.35, 3.75, 2.05); II: 15.55 (4.75, 5.05, 3.75, 2.00); III: 14.75 (4.50, 4.75, 3.50, 2.00); IV: 17.20 (5.00, 6.05, 4.05, 2.10). Carapace yellow, with black lateral margins. Abdomen greyish white. Epigyne: teeth long, subequal to the length of epigynal atrium; septum with weakly sclerotized posterior margin, about 0.3 times as long as wide; atrium with well delimited posterior margin, about 0.8 times as long as wide, about 1.7 times as long as septum, about 0.7 times as wide as septum; receptacles long, about 1.3 times as long as wide; copulatory opening indistinct; hoods indistinct (Fig. 16A–B).

##### Distribution.

**Figure 17.**
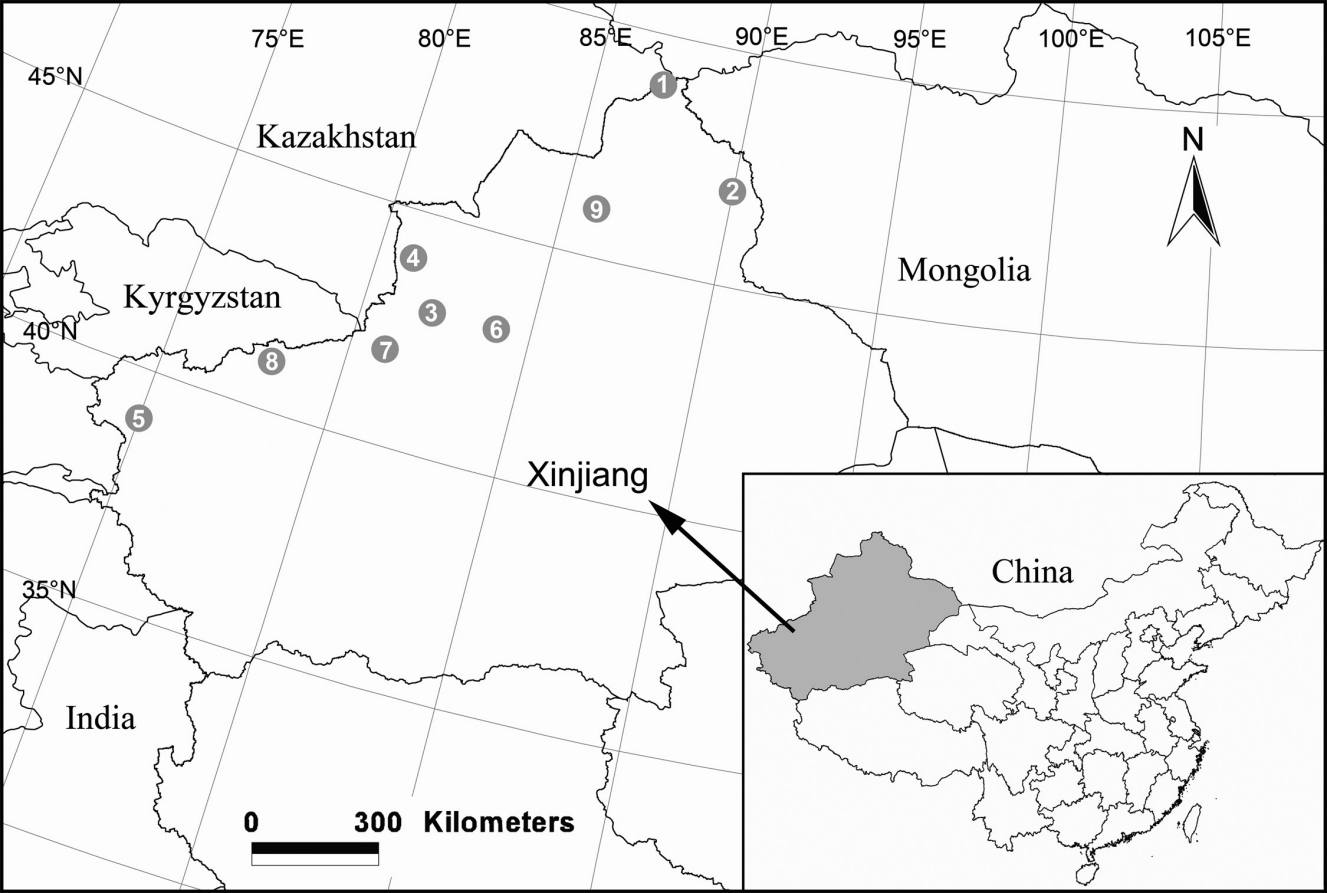
Localities of new *Pireneitega* species from Xinjiang, China. **1** *Pireneitega burqinensis* sp. n. **2** *Pireneitega fuyunensis* sp. n. **3** *Pireneitega gongliuensis* sp. n. **4** *Pireneitega huochengensis* sp. n. **5** *Pireneitega lini* sp. n. **6** *Pireneitega liui* sp. n. **7** *Pireneitega wensuensis* sp. n. **8** *Pireneitega wui* sp. n. **9** *Pireneitega yaoi* sp. n.

Known only from the type locality (Fig. 17).

## Supplementary Material

XML Treatment for Pireneitega

XML Treatment for Pireneitega burqinensis

XML Treatment for Pireneitega fuyunensis

XML Treatment for Pireneitega gongliuensis

XML Treatment for Pireneitega huochengensis

XML Treatment for Pireneitega lini

XML Treatment for Pireneitega liui

XML Treatment for Pireneitega wensuensis

XML Treatment for Pireneitega wui

XML Treatment for Pireneitega yaoi
